# Cell fusion induced senescence

**DOI:** 10.18632/aging.100670

**Published:** 2014-05-22

**Authors:** Hilah Gal, Valery Krizhanovsky

**Affiliations:** Department of Molecular Cell Biology The Weizmann Institute of Science 76100 Rehovot, Israel

Cellular senescence has long been established as a stable and irreversible growth arrest, which plays an important role in tumor suppression and tissue repair. It can be induced by various stimuli, including telomere shortening, oncogene activation and oxidative stress. We recently demonstrated that cell-cell fusion is a previously unidentified trigger for inducing cellular senescence [1]. This work was the first to demonstrate that fusion-induced senescent cells exist within a normally developed placenta, within an area where physiological cell-cell fusion occurs, thereby suggesting that cellular senescence plays a role in embryonic development.

Cell cycle arrest is a known consequence of cell-cell fusion that occurs in normal physiological processes, including tissue growth, immune regulation and embryonic development. However, endogenous and exogenous fusogenic proteins might lead to abnormal, so termed illicit, cell fusion. Normal and cancer cells undergoing cell-cell fusion triggered by such fusogens, such as the endogenous retroviral protein ERVWE1 or the measles virus (MV), display characteristics of cellular senescence [1]. The main molecular pathways of cellular senescence, the p53- and p16-pRb- pathways, are activated in fusion induced senescent (FIS) cells. Other components of the senescence phenotype, elevation in reactive oxygen species (ROS) and DNA damage, are also present in these cells. FIS cells express factors of the senescence-associated secretory phenotype (SASP) such as IL-6, IL-8, CCL5 and CXCL1. These factors might boost the interaction of FIS cells with their microenvironment, including the immune system. Therefore, FIS cells interact with the immune system, similarly to other types of senescent cells. To enhance the interaction with NK cells, FIS cells up-regulate the expression of ligands of activating NK cell receptor NKG2D. The combined secretion of SASP components and elevation of immune ligands by FIS cells support immune surveillance. Similar mechanisms are employed by the immune system to control the presence of senescent cells in cancer and in tissue damage restriction. The induction of the senescence program, when cells become illicitly fused in response to viral infection or aberrant expression of the fusogens, facilitates an immune response towards these cells. This may have evolved as a protection mechanism to prevent the spread of infection and cancer. Therefore, senescence induced by abnormal cell fusion might be a protective mechanism that limits propagation of damaged cells and facilitates their elimination by the immune system.

In physiological conditions within the placenta, ERVWE1 mediates the formation of the multinuclear syncytiotrophoblast, which serves as the maternal-fetal interface for the exchange of gas and nutrient supplies. The syncytiotrophoblasts exhibit multiple characteris-tics of cellular senescence. This suggests that FIS of syncytiotrophoblasts may play a physiological role in the development and proper functioning of the placenta [1]. There are several reasons as to why cell senescence may be important for the function of the placenta. Structurally, the flatted and enlarged morphology, typical to senescence cells, may help facilitate the transfer of nutrients in the maternal-fetal interface. The viability of the syncytiotrophoblast is essential to sustain pregnancy. Resistance of senescent cells to apoptosis might support syncytiotrophoblast viability despite the positive caspase-8 expression in this cell type. The expression of the anti-apoptotic DCR2, a common senescent marker, in the syncytiotrophoblast can further contribute to its viability. At the molecular level, the pRb pathway that regulates senescence in other settings is critical to the functionality of the placenta. Studies in mice have shown that knockdown of Rb pathway components in the placenta leads to placental malfunction and embryonic lethality. During pregnancy, the placenta relies on the secretion of cytokines for its proper regulation and function. The secretion of SASP-related cytokines may be necessary for maintaining the systemic inflammatory response within the placenta and promote a continuous interaction with the immune system. For example, IL-8 a well-established SASP component that is highly expressed in FIS cells, is essential for the proper functioning of the placenta. Further studies of different placental pathologies such as Intrauterine Growth Restriction (IUGR) and placenta accreta may shed new light on the role of FIS in the placenta.

It is plausible that FIS plays a dual role at different stages of life. During embryonic development, FIS plays a physiological role in placental development and functionality. In the adult organism the same mechanism is employed to protect the organism from the harmful effects of abnormal expression of fusogens and viral infections. Further research will reveal new insights into the role of FIS in physiological as well as pathophysiological settings.

Our work is supported by grants to V.K. from European Research Council and Marie Curie RG grants under the European Union's FP7, the Israel Science Foundation and the DKFZ-MOST program. V.K. is the incumbent of the Karl and Frances Korn Career Development Chair in Life Sciences.
